# Supplementary material for: Efficient enrichment cloning of TAL effector genes from Xanthomonas
Source: MethodsX. 2018 Sep 4;5:1027–32. doi: 10.1016/j.mex.2018.08.014 (PMC6138780; doi:10.1016/j.mex.2018.08.014)
Supplement: Supplementary file 1 [file mmc1.pdf]

Supplemental Table 1 | **In *silico* digestion analysis of *Xanthomonas oryzae* genome sequences**

**Clade**	**African *X. oryzae* pv. *oryzae***			**Asian *X. oryzae* pv. *oryzae***				***X. oryzae* pv. *oryzicola***	
**Origin**	**Mali**	**Burkina Faso**	**Cameroon**	**Philippines**	**Philippines**	**Korea**	**Japan**	**Philippines**	**Burkina Faso**
**Strain**	**MAI1**	**BAI3**	**AXO1947**	**PXO86**	**PXO99**[A]	**KACC 10331**	**MAFF 311018**	**BLS256**	**CFBP7342**
**Accession number**	pending	pending	CP013666	CP007166	CP000967	AE013598	AP008229	CP003057	CP007221
**Sequencing technology**	PacBio	PacBio	PacBio	PacBio	Sanger	Sanger	Sanger	Sanger	PacBio
**Reference**	unpublished	unpublished	Huguet-Tapia *et al.*, 2016, Genome Announc. 4: e01730-15	Booher *et al.*, 2015, Microb. Genom. 1:4	Salzberg *et al.*, 2008, BMC Genomics 9:204	Lee *et al.*, 2005, Nucleic Acids Res. 33:577-586	Ochiai *et al.*, 2005, Jpn. Agric. Res. Q. 39:275-287	Bogdanove *et al.*, 2011, J. Bacteriol. 193:5450-5464	Booher *et al.*, 2015, Microb. Genom. 1:4
**BamHI**	769	756	750	827	883	815	829	845	885
**ApaLI**	1671	1651	1637	1596	1662	1589	1587	1672	1693
**SfoI**	4986	4959	4881	4954	5223	4941	4980	4165	4564
**Total**[1]	102	102	103	108	123	105	109	145	157
**2-5 kb**[2]	15	15	15	23	28	21	23	31	27
**Number of *tal* gene BamHI fragments**[3]	9	9	9	16	18	13	16	27	23
**Number of *tal* genes**[4]	9	9	9	16 (2)	18 (1)	13	16 (1)	27 (1)	23 (1)
**Percentage (BamHI fragments)**	1.2	1.2	1.2	1.9	2.0	1.6	1.9	3.2	2.6
**Percentage (Total)**	8.8	8.8	8.7	14.8	14.6	12.4	14.7	18.6	14.6
**Percentage (2-5 kb)**	60.0	60.0	60.0	69.6	64.3	61.9	69.6	87.1	85.2

[1] Number of BamHI-fragments with BamHI-compatible overhangs predicted after combinational digestion with BamHI, ApalI and SfoI

[2] Number of BamHI-fragments in the size range of 2 to 5 kb with BamHI-compatible overhangs predicted after combinational digestion with BamHI, ApalI and SfoI

[3] Number of *tal* gene-related BamHI fragments predicted to be cloned following combinatorial digestion

[4] Number of *tal* genes in the TALomes as published or predicted, including truncated *tal* genes (in brackets)
