# Supplementary material for: Efficient enrichment cloning of TAL effector genes from Xanthomonas
Source: MethodsX. 2018 Sep 4;5:1027–32. doi: 10.1016/j.mex.2018.08.014 (PMC6138780; doi:10.1016/j.mex.2018.08.014)
Supplement: Supplementary file 2 [file mmc2.pdf]

Supplemental Table 2 | **Efficiency of cloning of *tal* gene BamHI fragments from *X. oryzae* strains MAI1 and BAI3 upon different enrichment treatments**

**Treatment**					**Number of different cloned *tal* gene BamHI fragments**
BamHI digestion	+	+	+	+	
Gel purification of 2-5 kb DNA fragments		+		+	
Double digestion with two counter-selection enzymes			+	+	
**Strain MAI1**	5 / 790[a]	5 / 630	10 / 324	28 / 89 & 34 / 136	9
	0.6%[b]	0.8%	3.1%	31.5% & 25.0%	
**Strain BAI3**	nt	nt	nt	57 / 212	8
				26.9%	

[a] Number of colonies with cloned *tal* gene BamHI fragments / number of analyzed colonies
[b] Percentage of colonies with cloned *tal* gene BamHI fragments
+ indicates that the sample was treated as indicated on the left.
nt, not tested
